# Dietary γ-Aminobutyric Acid (GABA) Improves Growth Performance and Increases Plasma Cortisol During Recovery from Short-Term Acute Thermal Exposure in Juvenile Rainbow Trout (*Oncorhynchus mykiss*)

**DOI:** 10.3390/ani16203324

**Published:** 2026-10-21

**Authors:** Suhyun Lee, Deni Aulia, Tugce Kilic, Haham Kim, Hyuncheol Jeon, Md Hashibur Rahman, Sooa Yoon, Abayomi Oladimeji Ogun, Seunghyung Lee

**Affiliations:** 1 Major of Aquaculture and Applied Life Sciences, Division of Fisheries Life Sciences, Pukyong National University, Busan 48513, Republic of Korea; su88842@naver.com (S.L.); damursalin@gmail.com (D.A.); tutti_91@hotmail.com (T.K.); haham7@naver.com (H.K.); conjp@naver.com (H.J.); hashibur@pukyong.ac.kr (M.H.R.); dbshelena@naver.com (S.Y.); abayomi.ogun@yahoo.com (A.O.O.); 2 Interdisciplinary Program of Marine and Fisheries Sciences and Convergent Technology, Pukyong National University, Busan 48513, Republic of Korea; 3 Feeds and Foods Nutrition Research Center, Pukyong National University, Busan 48547, Republic of Korea

**Keywords:** γ-aminobutyric acid (GABA), *Oncorhynchus mykiss*, growth performance, acute thermal exposure, cortisol

## Abstract

γ-aminobutyric acid (GABA) is a functional feed additive that may improve growth and stress resilience. The study evaluated the effects of dietary GABA on growth performance and recovery-phase physiological responses after short-term acute thermal exposure in juvenile rainbow trout. Two hundred twenty-five fish (18.9 ± 0.1 g) were fed five diets for 8 weeks: a control diet containing 68 mg/kg (GABA_68_) and diets with 108 (GABA_108_), 149 (GABA_149_), 183 (GABA_183_), and 218 (GABA_218_) mg/kg GABA. Fish were then subjected to short-term acute thermal exposure (20 °C for 2 h) followed by 2 h recovery at 15 °C. Dietary GABA significantly improved weight gain and specific growth rate, with the provisional model-based dietary GABA level for weight gain estimated to be approximately 198–210 mg/kg diet based on broken-line regression models, and increased feed and protein efficiency (*p* < 0.05). Fish fed the GABA183 and GABA218 diets had significantly higher plasma cortisol concentrations than those fed GABA68 and GABA108 when averaged across temperature treatments, with a significant temperature × GABA interaction (*p* < 0.05). During recovery, hepatic *hsp70α* and *hsp90βα* expression was significantly lower following 20 °C exposure than in fish maintained at 15 °C, whereas dietary GABA and its interaction with temperature had no significant effects on the stress-related genes analyzed. Dietary GABA improved growth performance and feed utilization; however, cortisol measured at a single recovery time point cannot distinguish a stronger stress response, delayed recovery, or altered cortisol clearance and does not demonstrate improved thermal tolerance or a faster recovery rate.

## Simple Summary

Temperature fluctuations pose a challenge to rainbow trout farming, highlighting the need for nutritional strategies that support fish growth and physiological function. This study investigated whether dietary gamma-aminobutyric acid, a naturally occurring compound involved in nerve signaling, could improve growth and influence responses to a brief increase in water temperature. Young rainbow trout were fed diets containing different levels of this compound for eight weeks. GABA supplementation improved growth and feed utilization, while higher dietary levels increased blood cortisol concentrations measured during recovery from exposure to warmer water. This increase could reflect a stronger stress response, slower recovery, or slower removal of cortisol from the blood. Because cortisol was measured at only one recovery time point, this study cannot distinguish these possibilities or demonstrate improved heat tolerance or faster recovery. These findings support the potential use of gamma-aminobutyric acid in feeds to improve rainbow trout production. Further research is needed to clarify its role in responses to temperature stress.

## 1. Introduction

Climate change has caused increased fluctuations in water temperatures, presenting significant challenges for cold-water aquaculture species, particularly salmonids [1]. Major mortality events attributed to temperature stress have been reported in salmonid aquaculture worldwide, with rising temperatures contributing to physiological stress, immune suppression, and reduced survival rates [2,3,4]. As poikilothermic organisms, fish are susceptible to environmental temperature changes, which directly impact critical physiological processes, including growth, enzyme activity, metabolic rates, immune function, overall health, and survival [4,5,6,7,8,9].

As a globally important cold-water aquaculture fish, rainbow trout (*Oncorhynchus mykiss*) has attracted widespread attention due to its high nutritional and economic value in aquaculture [10]. However, its susceptibility to temperature-induced stress presents a major challenge for sustainable production. The optimal temperature range for rainbow trout is 10–18 °C, with temperatures above this threshold disrupting metabolic balance, inducing oxidative stress, impairing immune function, and ultimately leading to increased mortality and reduced productivity [4]. For instance, when the temperature reaches above 20 °C, its appetite decreases, and growth becomes slow [10]. Once the temperature exceeds 24 °C, it ceases intake, with its life activities continuing to decline until death [4,10]. Given the projected increase in temperature variability due to climate change, developing effective strategies to enhance the temperature resilience of rainbow trout is critical for ensuring the sustainability of aquaculture operations.

Nutritional interventions have emerged as promising strategies to improve stress tolerance in aquaculture species [11]. One such intervention is the dietary supplementation of gamma-aminobutyric acid (GABA), a non-proteinogenic amino acid that acts as an inhibitory neurotransmitter in vertebrates. GABA plays a vital role in modulating neuronal excitability and stress responses, influencing growth, immune function, and overall physiological resilience in fish [12,13,14]. Several studies have shown that dietary GABA supplementation improves growth performance and metabolic efficiency in various aquaculture species, including olive flounder (*Paralichthys olivaceus*) [15], Nile tilapia (*Oreochromis niloticus*) [16,17], and juvenile grass carp (*Ctenopharyngodon idellus*) [18]. Despite the extensive research on GABA’s effects on growth and feed utilization, its potential role in mitigating temperature-induced stress in salmonids remains largely unexplored.

Therefore, this study evaluated the effects of dietary GABA supplementation on growth performance, feed utilization, whole-body composition, plasma biochemical indices, plasma and brain free amino acid profiles, growth-related gene expression, and physiological responses during recovery from acute thermal exposure in juvenile rainbow trout. Based on the measured parameters, this study aimed to determine the practical dietary GABA inclusion level for growth and to assess whether dietary GABA modulates metabolic and stress-related responses after short-term thermal exposure.

## 2. Materials and Methods

### 2.1. Ethics Statement

The research was carried out in strict compliance with the ethical guidelines and regulatory standards established by the Animal Care and Use Committee of Pukyong National University, Republic of Korea (Approval No. PKNUIACUC-2022-34, Approval date: 15 June 2022). All procedures involving fish including handling, rearing, sampling, and thermal stress challenges were designed to minimize discomfort, stress, and mortality. Husbandry conditions were monitored throughout the experimental period to ensure compliance with national and institutional standards for the ethical use of aquatic animals in research. Every effort was made to reduce the number of animals used and to refine experimental protocols to safeguard the welfare of the juvenile rainbow trout throughout the study.

### 2.2. Experimental Diets

**Figure 1:**
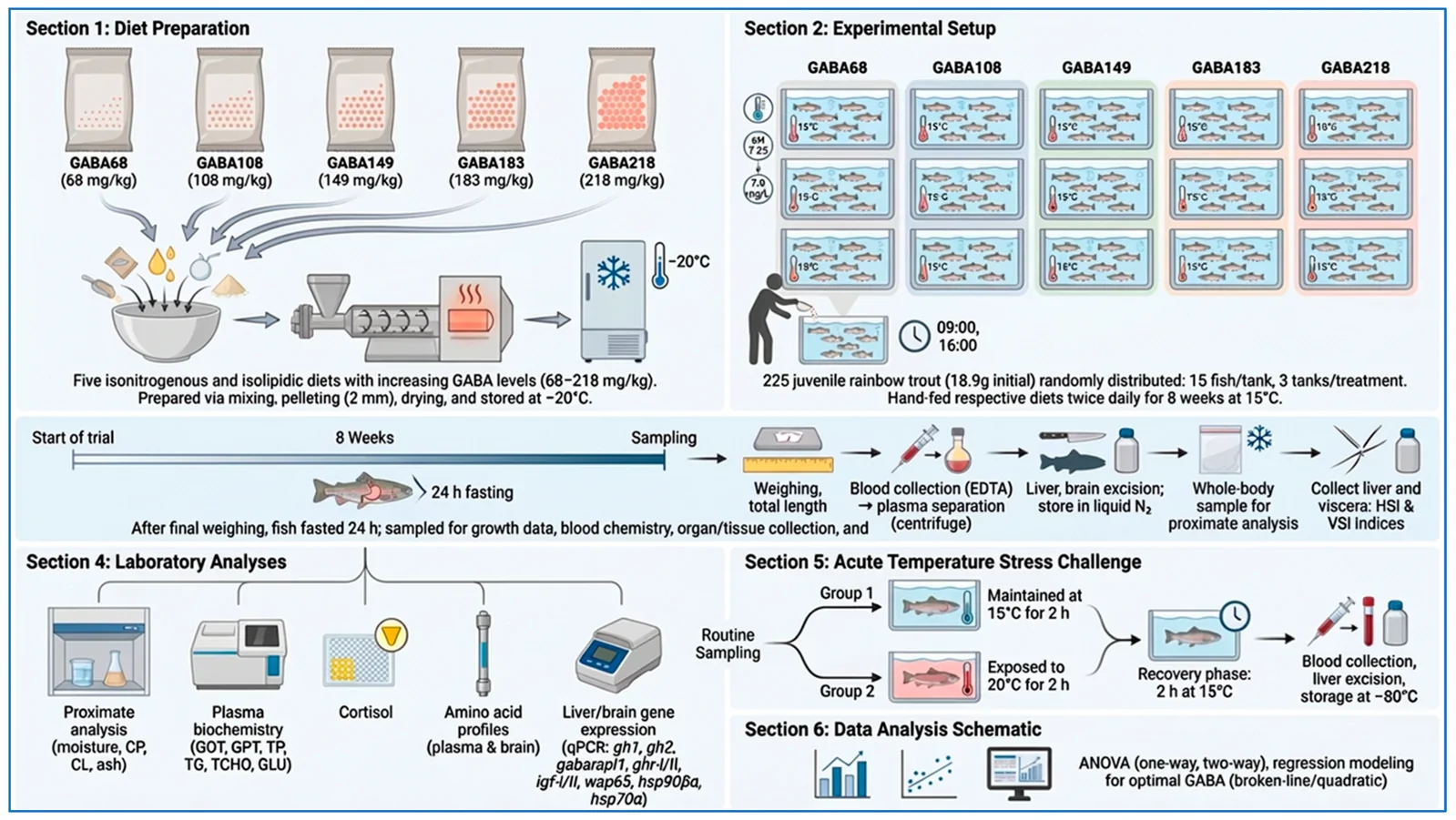
This schematic diagram illustrates the experimental design used to evaluate the effects of GABA-supplemented diets on fish growth, physiological stress responses, and metabolic changes. The growth trial consisted of an 8-week feeding period with diets containing different levels of GABA supplementation (68–217 mg/kg), followed by sampling for plasma metabolites, liver and brain gene expression, and whole-body nutrient composition. The acute thermal exposure and recovery test was then conducted, in which fish were either maintained at 15 °C or exposed to 20 °C for 2 h, followed by a 2 h recovery period at 15 °C. Plasma and liver samples were subsequently collected for metabolite, cortisol, and gene expression analyses.

To determine the optimal dietary supplementation level of γ-aminobutyric acid (GABA) for juvenile rainbow trout, five experimental diets were formulated to be isonitrogenous and isolipidic. GABA (≥99% purity; Sigma-Aldrich, St. Louis, MO, USA; catalog no. A2129; CAS no. 56-12-2) was used as the test additive and stored in a tightly closed container under dry conditions before diet preparation. A basal diet without supplemental GABA (GABA_68_) and four diets supplemented with 50 mg (GABA_108_), 100 mg (GABA_149_), 150 mg (GABA_183_), and 200 mg (GABA_218_) GABA per kilogram of diet were prepared, with α-cellulose substituted accordingly. Because ingredients such as fishmeal contained endogenous GABA, the basal diet (GABA_68_) also provided some naturally occurring GABA. Protein sources included anchovy fishmeal, squid liver powder, poultry by-product meal, soybean meal, tankage meal, and soy protein concentrate. Wheat flour and fish oil were used as carbohydrate and lipid sources, respectively. The schematic diagram of the experiment is illustrated in Figure 1.

Diet preparation and storage followed the procedures of Bai and Kim [19]. Briefly, dry ingredients were thoroughly mixed using a mixer (Model KB-108, Kimhill, Chia Yi, Taiwan). The mixture was moistened with water (~45% of total ingredient weight), and fish oil was gradually added until complete homogenization was achieved. The dough was pelleted through a 2-mm die (Baokyong Commercial Co., Busan, Republic of Korea), broken into small pellets by hand, spread on trays, and dried overnight in a room equipped with a dehumidifier. Dried pellets were individually bagged and stored at –20 °C until use in the feeding trial.

Proximate composition of the diets was analyzed at the Feeds and Foods Nutrition Research Center (FFNRC), Pukyong National University (Busan, Republic of Korea), following the Association of Official Analytical Chemists (AOAC) [20] methods. Moisture was determined by drying samples at 105 °C; ash by incineration at 550 °C using a muffle furnace (DAIHAN, WiseTherm^®^, Seoul, Republic of Korea); crude lipid by Soxhlet extraction using a Soxtec system 1046 (Tecator AB, Höganäs, Sweden); and crude protein by the Kjeldahl method (N × 6.25) using an Auto Kjeldahl system (B 324/435/412, BÜCHI Labortechnik AG, Flawil, Switzerland; 8-719/806, Metrohm AG, Herisau, Switzerland) after acid digestion. Total GABA concentrations in the finished diets were determined by high-performance liquid chromatography (HPLC) at FFNRC after overnight drying. The analyzed concentrations were 67.7, 108.0, 149.1, 183.1, and 217.8 mg/kg diet. GABA recovery during feed processing was not directly quantified because paired measurements before and after processing were not performed. The dried diets were stored at −20 °C until use; however, GABA stability during storage was not evaluated. The formulation and proximate composition of the five experimental diets are presented in Table 1.

### 2.3. Experimental Fish and Feeding Trial

An eight-week feeding trial was conducted at the Aquafeed Nutrition Laboratory, Pukyong National University (PKNU). Juvenile rainbow trout were obtained from An-il Fish Farm (Andong-si, Gyeongsangbuk-do, Republic of Korea) and transported to PKNU. Fish were stocked into rectangular tanks (77 L capacity; 30 L working volume) supplied with continuous freshwater flow (1.2 L/min) and aeration. Prior to the trial, fish underwent a two-week acclimation in an indoor semi-recirculating system and were fed a basal diet; all fish remained clinically healthy and lesion-free. The sex of the experimental fish was not determined.

For the feeding trial, 225 fish (initial body weight, IBW: 18.9 ± 0.1 g; mean ± SEM) were randomly allocated to 15 tanks (15 fish per tank). Tanks were then randomly assigned to one of five dietary treatments, with three replicate tanks per diet. The photoperiod during the feeding trial was maintained at 9 h light and 15 h dark. Water quality was monitored twice daily: temperature was maintained at 15.0 ± 1.0 °C using a Sun-Cool water temperature regulator (Model DH-2010, Daeil, Republic of Korea); pH was 7.35 ± 0.03; and dissolved oxygen (DO) was 7.02 ± 0.06 mg/L (means ± SEM). Fish were hand-fed their respective diets twice daily (09:00 and 16:00) at a daily ration of 2.0–2.5% body weight, adjusted based on observed feed intake. Every two weeks, the total number and biomass in each tank were recorded and rations recalculated accordingly. Tanks were siphoned after each feeding to remove uneaten feed and feces. Feed intake used to calculate FE and PER was determined by subtracting the quantity of recovered uneaten feed from the total feed supplied for each tank. Mortality was checked daily; any dead fish were removed immediately, and rations were recalculated to maintain the target feeding rate. Dead-fish biomass was not included in the biomass gain used to calculate FE and PER.

### 2.4. Sample Collection and Analyses

#### 2.4.1. Growth Performance

At the end of the feeding trial, fish were fasted for 24 h to allow complete gut evacuation. Final weighing was then conducted to record the weight and number of individuals in each tank. These data were used to calculate final body weight (FBW), weight gain (WG), specific growth rate (SGR), feed efficiency (FE), protein efficiency ratio (PER), and survival rate (SR). From each tank, six fish were randomly selected and anesthetized with 300 ppm phenoxyethanol for 5–10 min, while the remaining fish were returned to the tanks for the acute thermal exposure and recovery test. Anesthetized fish were individually measured for wet weight and total length to calculate condition factor (CF). Blood samples were collected from each fish using 1 mL syringes containing ethylenediaminetetraacetic acid (EDTA), pooled by tank into 3.0 mL blood collection tubes, and transferred to 1.5 mL microfuge tubes. Plasma was separated by centrifugation at 13,000 rpm for 5 min using a CF-10 centrifuge (Daihan Scientific, Seoul, Republic of Korea), immediately frozen in liquid nitrogen, and stored at −80 °C until analysis. Liver and brain tissues were excised, pooled by tank on aluminum foil, frozen in liquid nitrogen, and stored at −80 °C until analysis. After blood collection, three anesthetized fish per tank were placed in zipper bags and stored at −20 °C for whole-body proximate composition analysis. The remaining three fish were dissected to collect liver and viscera for the calculation of hepatosomatic index (HSI) and viscerosomatic index (VSI) as indicators of morphological changes in response to dietary treatments. Additional liver and brain samples were collected for amino acid profiling and gene expression analysis.

#### 2.4.2. Whole-Body Proximate Composition Analysis

Whole-body proximate composition was analyzed to evaluate the nutritional status of juvenile rainbow trout in response to dietary treatments. At the end of the feeding trial, fish samples were homogenized and freeze-dried for 48 h prior to analysis. The parameters measured included moisture, crude protein, crude lipid, and crude ash. Analytical procedures were identical to those used for the proximate composition of the experimental diets, as described above.

#### 2.4.3. Plasma Biochemical Parameters Analysis

Plasma samples were analyzed for biochemical parameters, including glutamic oxaloacetic transaminase (GOT), glutamic pyruvate transaminase (GPT), total protein (TP), triglycerides (TG), total cholesterol (TCHO), and glucose (GLU), using a chemical analyzer (Fuji DRI-CHEM NX500i, Fuji Photo Film Ltd., Tokyo, Japan). Plasma cortisol was measured using an enzyme-linked immunosorbent assay (ELISA) kit (CUSABIO, Houston, TX, USA; catalog no. CSB-E08487f). Standard reagents and plasma samples were processed according to the manufacturer’s instructions. Absorbance was determined at 450 nm with a microplate reader (AMR-100, Allsheng, Hangzhou, China). All measurements were performed in triplicate.

#### 2.4.4. Free Amino Acids Profile of Plasma and Brain Analysis

The free amino acid profiles of plasma and brain samples were determined by high-performance liquid chromatography (HPLC) at the Feeds and Foods Nutrition Research Center (FFNRC). Briefly, 0.3 g of tissue (solid) was homogenized using a hand-held homogenizer (Model D-130, Wiggens Co., Ltd., Beijing, China), after which 10 mL of HPLC-grade distilled water was added. The mixture was vortexed for 1 min at 5000 rpm using a multifunction vortex mixer (Maxshake™, Daihan Scientific, Wonju, Gangwon-do, Republic of Korea), followed by sonication for 20 min. Samples were then centrifuged at 4500 rpm for 10 min at 4 °C. A 0.7 mL aliquot of the supernatant was transferred to 1.5 mL microtubes, mixed with 0.7 mL of 7% sulfosalicylic acid (1:1, *v*/*v*), and incubated in the dark at 4 °C overnight. The mixture was centrifuged again at 4500 rpm for 10 min at 4 °C, and a 1 mL aliquot of the resulting supernatant was filtered through a 0.2 μm membrane filter before transfer to HPLC vials for analysis. For plasma samples, the same procedures were followed except that the homogenization step was omitted, as the sample was already liquid.

#### 2.4.5. Gene Expression of the Liver and Brain

Total RNA was extracted from liver and brain samples using the Hybrid-R^TM^ kit (GeneAll, Seoul, Republic of Korea) following the manufacturer’s instructions. RNA quantity and quality were assessed spectrophotometrically using a NanoDrop Lite Plus spectrophotometer (Thermo Fisher Scientific, Waltham, MA, USA). Complementary DNA (cDNA) was synthesized using the PrimeScript™ 1st Strand cDNA Synthesis Kit (Takara Bio, Kusatsu, Shiga, Japan) according to the manufacturer’s protocol. The cDNA samples were stored at −20 °C until use for quantitative PCR (qPCR). qPCR was performed to evaluate mRNA expression levels of *ghr-I*, *ghr-II*, *igf-I*, *igf-II*, *gh1*, *gh2*, *gabarapl1*, *wap65-1*, *wap65-2*, *hsp90βα*, and *hsp70α*. *β-actin* served as the internal control (housekeeping gene). The stability of *β-actin* expression across dietary treatments was not independently validated; this represents a limitation of the relative expression analysis. Reactions were carried out on a StepOne Real-Time PCR system (Applied Biosystems, Waltham, MA, USA) using TB Green^®^ Premix Ex Taq™ II (Tli RNaseH Plus; Takara Bio, Kusatsu, Japan). Primer sequences for all target genes are shown in Table 2. The thermal cycling program consisted of an initial denaturation at 95 °C for 30 s, followed by 40 cycles of denaturation at 95 °C for 5 s and annealing/extension at 60 °C for 30 s. A melting curve analysis was performed at the end of the amplification to confirm product specificity. For each target gene, qPCR reactions were performed in triplicate. Relative mRNA expression levels were normalized to *β-actin* and calculated using the 2^−ΔΔCt^ method. The mean Ct value from technical triplicates was used for each biological replicate, and ΔCt values were calculated by subtracting the Ct value of *β-actin* from that of each target gene. For statistical analysis, ΔCt values were used, whereas 2^−ΔΔCt^ fold-change values are presented in the tables and figures for biological interpretation. Data analysis was performed with StepOne Software version 2.0 (Applied Biosystems, Waltham, MA, USA).

#### 2.4.6. Acute Thermal Exposure and Recovery Test

An acute thermal exposure and recovery test was conducted 24 h after the final weighing to evaluate short-term physiological responses during the recovery phase. From each replicate tank, six healthy fish were randomly selected and divided into two groups. Three fish were maintained at 15 °C, which was the rearing temperature used during the feeding trial, while the other three fish were abruptly exposed to 20 °C for 2 h. Water temperature was controlled using an artificial thermostat. After the 2 h exposure period, fish from both temperature groups were transferred to separate tanks maintained at 15 °C for a 2 h recovery period. Fish from each original dietary replicate tank were exposed separately under each temperature condition, and samples collected from fish originating from the same replicate tank were pooled. Therefore, the original replicate tank, rather than individual fish, was used as the biological experimental unit for statistical analysis. Each dietary treatment therefore had three biological tank replicates under each temperature condition. The three fish sampled within each tank–temperature group were pooled to provide one biological observation. Therefore, individual fish and technical replicate measurements did not increase the biological sample size.

After recovery, fish were anesthetized, and plasma was collected for metabolite and cortisol analyses. Liver samples were also excised for gene expression analysis. This test was designed to assess recovery-phase physiological responses after short-term acute thermal exposure, rather than chronic thermal tolerance, survival capacity, or long-term heat-stress resistance. The procedures for anesthesia, blood collection, and liver sampling were identical to those described previously. Cortisol and hepatic gene expression were measured only at the end of the 2 h recovery period. This single sampling point cannot characterize response dynamics or distinguish a stronger cortisol response, altered cortisol clearance, or delayed recovery. The short-term exposure protocol does not reproduce chronic or fluctuating thermal conditions encountered in aquaculture.

### 2.5. Calculations and Statistical Analysis

#### 2.5.1. Determination of Growth Parameters and Body Indices

At the end of the 8-week feeding trial, all fish in each tank were counted and bulk-weighed 24 h after the last feeding to evaluate growth performance, feed utilization, and survival. The measured growth and feed utilization parameters included weight gain (WG), specific growth rate (SGR), feed efficiency (FE), and survival rate (SR). In addition, three fish were randomly selected from each tank, anesthetized with 2-phenoxyethanol (300 ppm; Sigma-Aldrich, St. Louis, MO, USA), and individually measured for hepatosomatic index (HSI), and viscerosomatic index (VSI). For FE and PER, biomass gain was calculated as final surviving tank biomass minus initial tank biomass, without correction for dead-fish biomass. The growth performance and morphological indices were calculated using the following equations:

WG (%) = [final weight (g) − initial weight (g)]/intial weight (g) × 100

SGR (%) = [ln final weight (g) − ln intial weight (g)]/days of trial × 100

FE (%) = [final weight (g) − initial weight (g)]/feed consumed (g) × 100

$$\mathrm{PER}=\frac{\mathrm{Weight}\mathrm{gain}(g)}{\mathrm{Protein}\mathrm{intake}(g)}$$

SR (%) = final number of fish/initial number of fish × 100

CF = fish weight (g)/total length (cm)^3^ × 100

HSI (%) = liver weight (g)/fish weight (g) × 100

VSI (%) = viscera weight (g)/fish weight (g) × 100

#### 2.5.2. Determination of the Optimum Inclusion Level

A dose–response regression analysis was performed to estimate provisional model-based dietary GABA levels for weight gain (WG) in juvenile rainbow trout. The actual analyzed GABA concentrations in the diets were used as the independent variable (x), and tank-level WG was used as the response variable (y). Four regression models were evaluated: one-slope straight broken-line model, two-slope straight broken-line model, quadratic broken-line model, and quadratic model, following Lee et al. [32,33].

For the one-slope straight broken-line model, the response changes linearly up to a breakpoint and then reaches a plateau:

y = β0 + β1(R − x), if x < R

y = β0, if x ≥ R

For the two-slope straight broken-line model, the response changes slope after the breakpoint:

y = β0 + β1(R − x), if x < R

y = β0 + β2(x − R), if x ≥ R

For the quadratic broken-line model, the response follows a quadratic curve before reaching a plateau:

y = β0 + β1(R − x)^2^, if x < R

y = β0, if x ≥ R

For the quadratic model, the response was described as:

y = β0 + β1x + β2x^2^

Here, R denotes the breakpoint, and β_0_, β_1_, and β_2_ denote model coefficients. For the quadratic model, a candidate maximum was calculated as −β_1_/(2β_2_) only when the fitted curve was concave and its vertex occurred within the tested dietary range. For the broken-line models, R represents the estimated point at which the fitted response changes slope or reaches a plateau, depending on the model.

Model fit was evaluated using the adjusted coefficient of determination (adjusted R^2^) and corrected Akaike information criterion (AICc), with higher adjusted R^2^ and lower AICc indicating better fit. A provisional dietary GABA range was interpreted using model-fit criteria and the plateau responses represented by the one-slope and quadratic broken-line models. This range represents breakpoint estimates from two different models, rather than a 95% confidence interval or a definitively established dietary requirement.

#### 2.5.3. Statistical Analysis

Data from the eight-week feeding trial were analyzed using one-way ANOVA to evaluate the effects of dietary treatments on growth performance, body indices, and whole-body composition. When significant differences were detected, Tukey’s honestly significant difference (HSD) test was applied for post hoc comparisons among treatment groups. Exploratory linear and quadratic trend analyses were performed to characterize dose-related patterns across dietary GABA levels. These analyses complemented the assessment of overall dietary differences using one-way ANOVA. Significant trends detected without a significant overall ANOVA were interpreted cautiously and were not considered evidence of significant pairwise differences. Linear and quadratic trends were evaluated to assess dose-related patterns across dietary GABA levels. These tests assessed specific response patterns, whereas overall differences among dietary groups were assessed using one-way ANOVA. Significant trends detected in the absence of a significant overall ANOVA were interpreted cautiously and were not considered evidence of significant pairwise differences. Data from the acute thermal exposure and recovery test, including plasma metabolites, cortisol, and hepatic gene expression, were analyzed using a linear mixed-effects model. Dietary GABA, temperature, and their interaction were specified as fixed effects, and original tank nested within dietary treatment was included as a random effect to account for the shared source tank of samples evaluated under both temperature conditions. Gene expression analyses were performed on ΔCt values. When no significant interaction was observed, the main effects of GABA supplementation and temperature were tested separately. All statistical analyses were conducted using SAS software (version 9.4, SAS Institute Inc., Cary, NC, USA). The assumptions of normality and homogeneity of variance were assessed using the Shapiro–Wilk and Levene’s tests, respectively. Differences were considered statistically significant at *p* < 0.05. Data are presented as means ± standard error of the mean (SEM).

## 3. Results

### 3.1. Growth Performance

**Figure 2:**
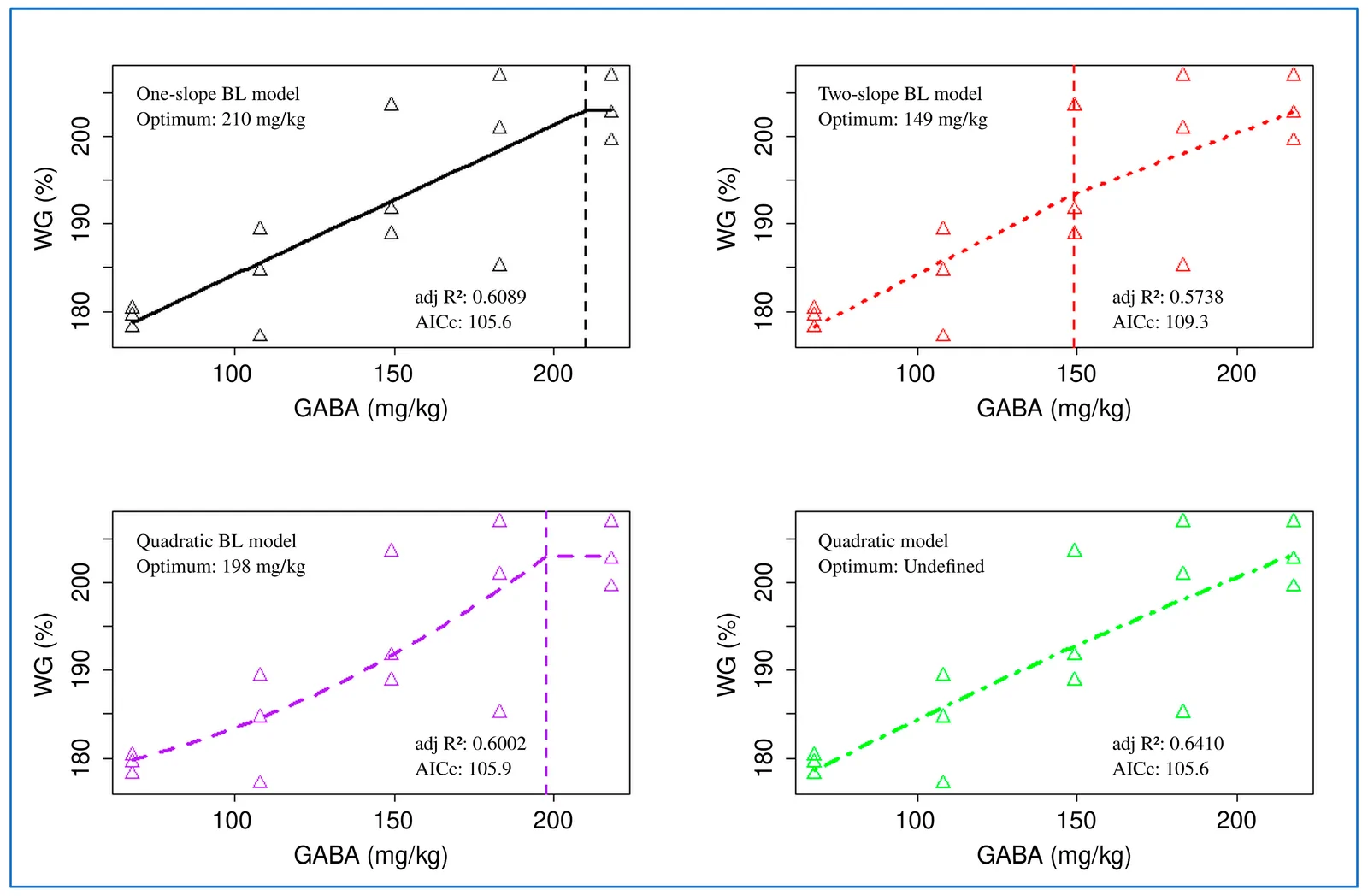
The optimal dietary inclusion level of γ-aminobutyric acid (GABA) for weight gain (WG) in juvenile rainbow trout using four regression models. The one-slope broken-line (BL) model estimated an optimum of 210 mg/kg diet, whereas the two-slope BL and quadratic BL models estimated optimum levels of 149 and 198 mg/kg diet, respectively. The quadratic model did not provide a clearly defined optimum within the tested dietary range. Model fit was evaluated using the adjusted coefficient of determination (adjusted R^2^) and corrected Akaike information criterion (AICc). A provisional model-based range of approximately 198–210 mg/kg diet was derived from the one-slope and quadratic broken-line models, which had similar AICc values.

Table 3 illustrates the effects of dietary GABA inclusion on the growth performance of juvenile rainbow trout over eight weeks. Survival rates remained high, exceeding 97% across all groups, with no significant differences (*p* > 0.05). Dietary GABA significantly influenced final body weight (FBW), weight gain (WG), and specific growth rate (SGR). The highest numerical FBW, WG, and SGR values were observed in the GABA218 group, and significant positive linear trends were detected for these parameters (*p* < 0.05). Specifically, FBW was significantly higher in the GABA149 and GABA218 groups than in the GABA68 and GABA108 groups, whereas the GABA183 group showed an intermediate value. For WG and SGR, the GABA218 group showed significantly higher values than the GABA68 and GABA108 groups, while the GABA149 and GABA183 groups showed intermediate values that were not significantly different from either the lower or higher GABA groups. These results indicate that dietary GABA improved growth performance in juvenile rainbow trout, with the strongest growth response observed in fish fed the GABA218 diet. Regression analysis further estimated the provisional model-based dietary GABA level for WG to be approximately 198–210 mg/kg diet, depending on the model used (Figure 2).

In terms of feed efficiency (FE), the GABA_218_ group showed significantly higher values than the GABA_68_ group (*p* < 0.05), although there were no significant differences among the other test diets. Similarly, the protein efficiency ratio (PER) was significantly higher in the GABA_218_ group compared to the GABA_68_ and GABA_108_ groups, with no significant differences between the GABA_149_ and GABA_183_ groups. Regarding survival rate (SR), condition factor (CF), hepatosomatic index (HSI), and viscerosomatic index (VSI), no significant differences were observed, and both linear and quadratic trends were not significant (*p* > 0.05).

The estimated dietary GABA levels for weight gain were 210, 149, and 198 mg/kg diet using the one-slope, two-slope, and quadratic broken-line models, respectively. The highest adjusted R^2^ was obtained with the quadratic model (0.6410; AICc = 105.6), but no defined optimum was obtained within the tested dietary range. Among the broken-line models, the highest adjusted R^2^ and lowest AICc were obtained with the one-slope model (0.6089 and 105.6), followed by the quadratic broken-line model (0.6002 and 105.9). The lowest adjusted R^2^ and highest AICc were obtained with the two-slope model (0.5738 and 109.3). Therefore, approximately 198–210 mg/kg diet was interpreted as a provisional model-based range derived from the two plateau models with similar AICc values. The range of 198–210 mg/kg diet represents the breakpoint estimates obtained from two different regression models and is not a 95% confidence interval. It should therefore be interpreted as a provisional model-based range rather than a precisely established dietary requirement.

### 3.2. Whole-Body Proximate Composition

The whole-body proximate composition of juvenile rainbow trout fed the experimental diets for eight weeks is presented in Table 4. Moisture content was significantly lower in the GABA183 group than in the GABA68 group (*p* < 0.05). Intermediate values were observed in the GABA108, GABA149, and GABA218 groups, which did not differ significantly from either GABA68 or GABA183. No significant differences among dietary groups were observed for crude protein, crude lipid, or crude ash contents (*p* > 0.05). However, trend analysis showed a significant linear response for moisture and crude lipid contents and a significant quadratic response for crude ash content. These results indicate that, apart from the moisture difference between GABA68 and GABA183, no significant pairwise differences were detected in whole-body composition. The significant trend results should be interpreted cautiously and do not establish differences between individual dietary groups.

### 3.3. Plasma Biochemical Parameters

The plasma biochemical parameters of juvenile rainbow trout fed the experimental diets for eight weeks are shown in Table 5. There were no significant differences among dietary groups in plasma glutamic oxaloacetic transaminase, glutamic pyruvic transaminase, total protein, triglyceride, total cholesterol, or glucose levels (*p* > 0.05). However, trend analysis showed a significant quadratic response for glutamic pyruvic transaminase and a significant linear response for triglyceride. These results suggest that dietary GABA supplementation did not markedly alter plasma biochemical parameters under normal rearing conditions, although selected trend responses were detected for GPT and TG.

### 3.4. Free Amino Acid Profile of Plasma and Brain

The free amino acid profiles of plasma and brain in juvenile rainbow trout fed the experimental diets for eight weeks are presented in Table 6 and Table 7, respectively. No significant dietary differences were detected in the plasma profile except for taurine. Fish fed the GABA108 diet exhibited significantly higher plasma taurine levels (114 ± 10 ppm) than those fed the GABA68 (85.4 ± 1.3 ppm) and GABA149 (82.2 ± 2.6 ppm) diets (*p* < 0.05). Intermediate values were observed in the GABA183 and GABA218 groups, which did not differ significantly from the other dietary groups. No significant linear or quadratic trend was detected for plasma taurine (*p* > 0.05). No significant dietary differences were detected in any measured brain free amino acids (*p* > 0.05).

### 3.5. Gene Expression

The results of the relative gene expression levels in the brain and liver of juvenile rainbow trout fed different experimental diets for eight weeks are shown in Table 8 and Table 9, respectively. There were no significant differences in the expression of the *gh1*, *gh2*, and *gabarapl1* genes in the brains of fish fed the experimental diets (*p* > 0.05). The expression of the *ghr-I* and *igf-II* genes in the liver showed no significant differences among fish fed the five experimental diets (*p* > 0.05). Hepatic *ghr-II* expression was significantly lower in fish fed GABA108 than in those fed GABA68 and GABA218, whereas intermediate values were observed in the GABA149 and GABA183 groups. Hepatic *igf-I* expression was significantly higher in fish fed GABA218 than in those fed GABA108, whereas intermediate values were observed in the remaining dietary groups (*p* < 0.05). However, there were no significant differences among fish fed GABA68, GABA149, GABA183, and GABA218 diets (*p* > 0.05).

### 3.6. Acute Thermal Exposure and Recovery Test

The effects of the acute thermal exposure and recovery test on plasma biochemical parameters and cortisol levels in juvenile rainbow trout fed the experimental diets are presented in Table 10 and Supplementary Figure S1. No significant interactions between temperature and GABA were observed for plasma glutamic oxaloacetic transaminase, glutamic pyruvic transaminase, total protein, triglyceride, total cholesterol, or glucose levels (*p* > 0.05). Similarly, temperature had no significant main effect on these plasma biochemical parameters (*p* > 0.05). However, dietary GABA had a significant main effect on total protein, with the GABA183 group showing a significantly higher value than the control group (*p* < 0.05).

For cortisol, a significant interaction effect between temperature and dietary GABA was observed (*p* < 0.05). The GABA68 group maintained at 15 °C exhibited the lowest cortisol level, whereas the GABA218 group exposed to 20 °C showed the highest cortisol concentration. The GABA218 group exposed to 20 °C had the highest mean cortisol concentration and differed significantly from all groups except the GABA183 group exposed to 20 °C. A significant main effect of temperature was also observed (*p* < 0.05), with cortisol levels being significantly higher at 20 °C than at 15 °C. Likewise, a significant main effect of dietary GABA was observed (*p* < 0.05), with the GABA183 and GABA218 groups showing significantly higher cortisol levels than the control (GABA68) and GABA108 groups. However, there were no significant differences between the GABA108 and GABA149 groups, nor among the GABA149, GABA183, and GABA218 groups. The box plot further illustrates the temperature-dependent increase in cortisol, particularly in fish fed higher dietary GABA levels (Supplementary Figure S1G). These results indicate that dietary GABA influenced plasma cortisol concentration during the recovery phase after short-term acute thermal exposure.

**Figure 3:**
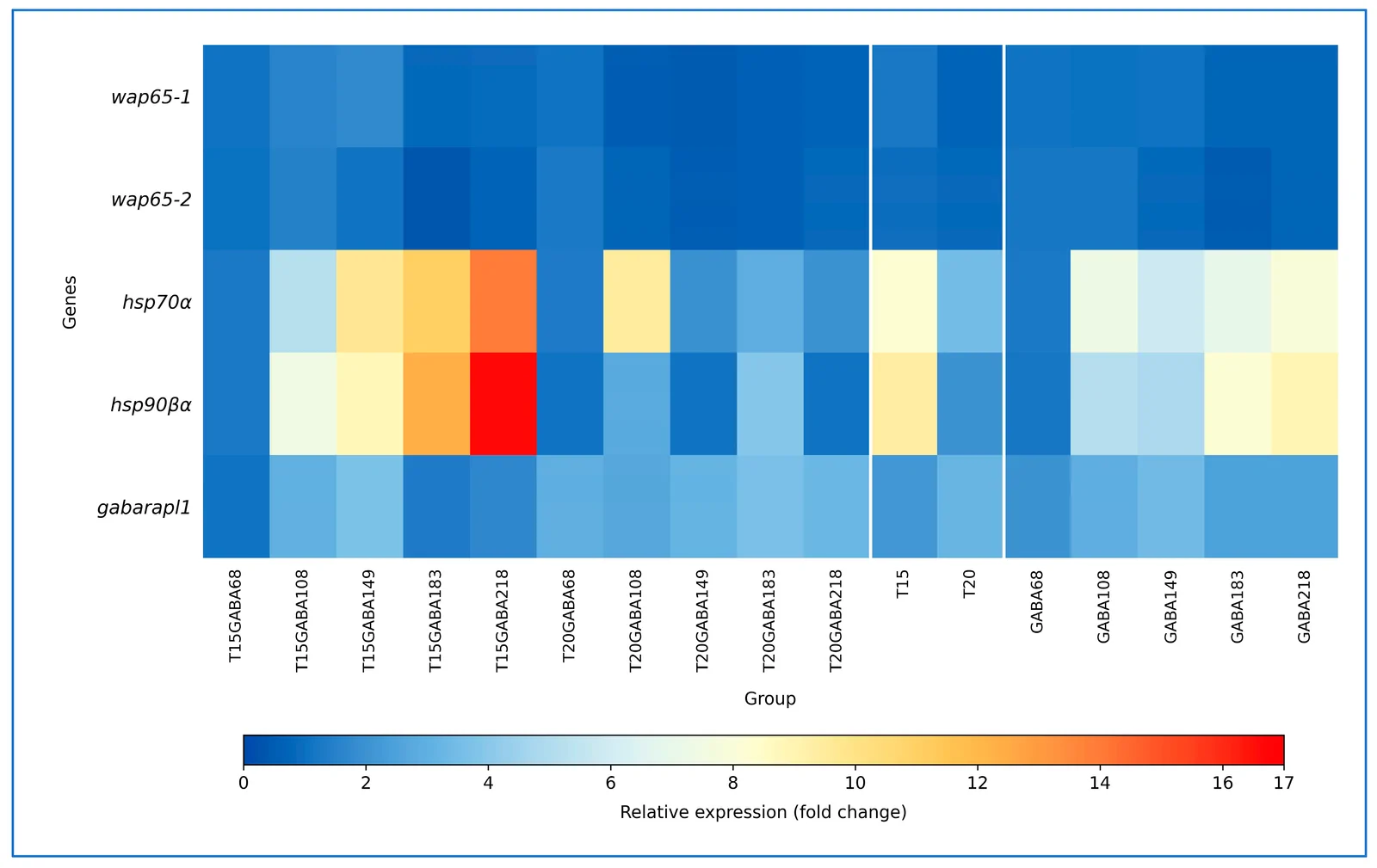
Heatmap of hepatic stress-related gene expression in juvenile rainbow trout after the acute thermal exposure and recovery test. Relative mRNA expression levels of *wap65-1*, *wap65-2*, *hsp70α*, *hsp90βα*, and *gabarapl1* were measured in the liver after fish were maintained at 15 °C or exposed to 20 °C for 2 h, followed by 2 h recovery at 15 °C. Columns represent the interactive treatment groups, temperature main effects, and dietary GABA main effects. Color intensity indicates relative expression level, with blue representing lower expression and red representing higher expression. Expression values were normalized to β-actin, and T15GABA68 was used as the control group.

The relative hepatic expression levels of stress-related genes in juvenile rainbow trout after the acute thermal exposure and recovery test are presented in Table 11 and Figure 3. Temperature significantly affected *hsp70α* (*p* = 0.0195) and *hsp90βα* (*p* = 0.0004) expression, with lower mean expression in fish exposed to 20 °C for 2 h followed by 2 h recovery at 15 °C than in fish maintained at 15 °C. Temperature had no significant effect on *wap65-1*, *wap65-2*, or *gabarapl1* expression (*p* > 0.05). Neither dietary GABA nor the temperature × GABA interaction significantly affected any of the genes analyzed (*p* > 0.05). The heatmap further illustrates variation in the relative expression of these genes across treatment groups (Figure 3); statistical significance is reported in Table 11.

## 4. Discussion

This study examined the effects of dietary gamma-aminobutyric acid (GABA) supplementation on growth performance, feed utilization, physiological status, and recovery-phase responses after short-term acute thermal exposure in juvenile rainbow trout (*Oncorhynchus mykiss*). Growth parameters, including final body weight (FBW), weight gain (WG), specific growth rate (SGR), protein efficiency ratio (PER), and feed efficiency (FE), showed a significant linear increase with higher GABA supplementation, with the GABA_218_ group exhibiting significantly superior growth performance compared to the control (GABA_68_), indicating enhanced nutrient utilization and growth efficiency. These findings align with previous reports on olive flounder (*Paralichthys olivaceus*) [15], Nile tilapia (*Oreochromis niloticus*) [16,17], juvenile grass carp (*Ctenopharyngodon idellus*) [18], and juvenile Pacific white shrimp (*Litopenaeus vannamei*) [34], which similarly demonstrated improved growth performance with GABA supplementation. Regression analysis of weight gain in the present study estimated the provisional model-based dietary GABA level to be approximately 198–210 mg/kg diet, based on the one-slope broken-line and quadratic broken-line models. This range is comparable to the 237 mg/kg reported for olive flounder [15], but higher than the 87.5 mg/kg and 88.6 mg/kg reported for grass carp [18] and Chinese mitten crab [35], respectively. However, only five dietary GABA levels were evaluated, limiting the precision with which the response shape and breakpoint could be characterized. A plateau was assumed in the one-slope and quadratic broken-line models, whereas a second slope was permitted after the breakpoint in the two-slope model. The estimated range of 198–210 mg/kg diet should therefore be interpreted cautiously, and additional dietary levels around and above this range are needed for confirmation. Despite the positive effects on growth performance, dietary GABA supplementation had no significant impact on survival rate (SR), condition factor (CF), hepatosomatic index (HSI), or viscerosomatic index (VSI), consistent with previous studies on olive flounder [13,15], suggesting that while GABA enhances growth efficiency, it does not influence body morphology or organ indices in this species.

Furthermore, our analysis of whole-body proximate composition revealed that dietary GABA inclusion had minimal effects on macronutrient composition, with only moisture content showing significant reduction in the GABA183 group compared to the control. This observation aligns with those of Li et al. [36] and Barido et al. [37] who reported that dietary GABA inclusion reduced muscle moisture content in pharaoh cuttlefish (*Sepia pharaonis*) and Hanwoo steers (*Bos taurus coreanae*), respectively. Although intracellular water content has been associated with muscle strength and functional capacity [38], these outcomes were not measured in the present study. The observed whole-body moisture difference therefore does not establish altered water retention or muscle quality. Similarly, the significant trends in crude lipid and ash, without significant overall dietary differences, do not establish changes in lipid deposition or mineral metabolism. On the contrary, in *Labeo rohita* fingerlings and juvenile *Litopenaeus vannamei*, GABA supplementation did not affect whole-body composition [39,40], suggesting that the influence of GABA in influencing moisture levels may depend on the type of species being reared.

Monitoring free amino acid profiles can provide insights into nutrient availability, metabolic utilization, and physiological responses to dietary interventions [14,41]. In the present study, no significant dietary differences were detected in the measured plasma or brain free amino acid (FAA) profiles, except for plasma taurine. These results provide limited evidence of changes in the measured FAA profiles following dietary GABA supplementation. Dietary protein deficiency has been reported to lead to the utilization of both essential and non-essential amino acids to meet metabolic demands [13], which can alter the free amino acid profile [42]. Additionally, plasma amino acid profiles in rainbow trout have been shown to be influenced by different dietary nutrient conditions [43]. However, the absence of significant differences in the present study does not establish that amino acid homeostasis was unaffected or identify the mechanisms maintaining FAA concentrations.

Plasma taurine levels were significantly affected by dietary GABA supplementation, with the highest value observed in the GABA108 group. However, no significant linear or quadratic trend was detected, indicating that the response was not clearly dose-dependent. Although non-linear and biphasic responses have been described in other biological systems [44,45] and in animals or aquatic models exposed to bioactive compounds or environmental stressors [46,47,48,49,50], such interpretations are not supported by the present taurine data. This isolated difference should therefore be interpreted cautiously rather than as evidence of a consistent effect on taurine metabolism. Further investigation is needed to determine whether it reflects altered taurine metabolism or normal biological variation [51]. Overall, dietary GABA had limited effects on the measured FAA profiles, and no significant dietary differences were detected in brain FAA concentrations.

Haematological and blood biochemistry parameters are valuable tools increasingly used in aquaculture studies to assess fish metabolism and overall health status [52]. In the present study, we investigated the effects of dietary GABA supplementation on plasma metabolites after the termination of the feeding trial. These metabolites included key indicators of liver function, including glutamate oxaloacetate transaminase (GOT) and glutamate pyruvate transaminase (GPT). Additionally, we analyzed total protein (TP), which plays a vital role in immune response and growth; triglycerides (TG), a major energy reserve; total cholesterol (TCHO), essential for cell membrane integrity and steroid hormone synthesis; and glucose (GLU), a primary source of metabolic energy. Our observations showed that dietary GABA supplementation had no significant effects on these metabolites. However, TG levels exhibited a significant linear trend, increasing with higher GABA inclusion, while GPT levels showed a significant quadratic trend, initially rising before declining at higher inclusion levels. These findings suggest that while GABA does not markedly alter key metabolic indicators, it may subtly influence lipid metabolism and liver enzyme activity. Similar findings have been reported in previous studies. Bae et al. [53] observed no significant changes in aspartate aminotransferase (AST/GOT), alanine aminotransferase (ALT/GPT), total protein (TP), and glucose (GLU) levels in olive flounder fed GABA-supplemented diets. Likewise, Gunathilaka et al. [54] found that plasma triglyceride (TG) levels in juvenile red seabream (*Pagrus major*) were unaffected by dietary GABA supplementation. Additionally, Temu et al. [16] reported no significant effects of GABA supplementation on plasma total cholesterol (TCHO) levels in Nile tilapia. Although significant linear and quadratic trends were observed for plasma TG and GPT, respectively, no significant overall differences were detected among dietary groups. These findings should be interpreted cautiously, and further investigation is needed to clarify their relevance to lipid metabolism and liver enzyme activity.

Metabolic and physiological processes in fish are governed by complex molecular mechanisms, particularly within key organs such as the brain and liver, where cells integrate metabolic information into core molecular processes like transcription to adapt to environmental changes [55]. By examining gene expression in these tissues, we can gain valuable insights into how dietary interventions influence biological functions. In this study, we assessed the effects of dietary GABA supplementation on metabolic pathways in the brain and liver of juvenile rainbow trout before exposing them to stress. Specifically, we focused on genes involved in growth regulation, hormonal signaling, and metabolic processes. In the brain, we analyzed growth hormone 1 (*gh1*), growth hormone 2 (*gh2*), and GABA receptor-associated protein like 1 (*gabarapl1*), while in the liver, we investigated growth hormone receptor 1 (*ghr-I*), growth hormone receptor 2 (*ghr-II*), insulin-like growth factor 1 (*igf-I*), and insulin-like growth factor 2 (*igf-II*). Significant dietary differences were detected in hepatic *ghr-II* and *igf-I* expression, with lower values observed in the GABA108 group. However, a consistent increase across dietary GABA levels was not observed. These differences therefore do not establish a dose-dependent stimulation of growth-related pathways, and the observed growth improvement cannot be attributed directly to these transcript measurements. Consistent with our results, Lee et al. [56] also found no significant alterations in the expression of related genes in olive flounder following GABA supplementation. These findings indicate that improved growth performance was not accompanied by consistent dietary responses across the growth-related genes analyzed. In contrast, Ruenkoed et al. [17] reported that GABA supplementation upregulated *ghr-II* and *igf-I* expression, while Kumar et al. [40] observed significant changes in *igf-I* mRNA expression in *Labeo rohita*. Similarly, Athapaththu et al. [57] found that GABA treatment in zebrafish larvae (*Danio rerio*) resulted in increased growth, attributed to upregulation.

Gene expression is regulated by multiple factors, making it difficult to isolate the specific determinants of transcriptional variation [58,59]. Understanding how these factors interact to influence organismal phenotypes remains a significant challenge [60]. The discrepancies observed across studies may be attributed to GABA’s dual mode of action, which operates both centrally through the nervous system and directly at the pituitary level. The dual sites of GABA action likely account for its excitatory and inhibitory effects, influencing hormone secretion dynamics. The resulting physiological outcomes may vary depending on the specific site of action within the hypothalamic-pituitary unit and the hormonal environment at the time [61]. These findings highlight the need for further research to explore species-specific and context-dependent mechanisms regulating the physiological effects of dietary GABA.

Temperature stress in aquaculture affects key physiological processes, including nutrient uptake, hormonal regulation, and metabolism. In this study, we investigated how dietary GABA supplementation influences plasma metabolite profiles during the recovery phase following a two-hour stress exposure. Given the critical roles of liver function and metabolic homeostasis in stress resilience, we measured changes in GOT (AST) and GPT (ALT) as indicators of hepatic function, TP as a marker of protein metabolism, and TG, TCHO, and GLU to assess shifts in lipid metabolism and energy balance. To further elucidate the physiological stress response, we quantified cortisol (CORT) levels and analyzed the expression of key stress-related genes, including *wap65-1* and *wap65-2* (temperature acclimation genes), *hsp70* and *hsp90βα* (genes encoding heat shock proteins), and *gabarapl1* (associated with GABAergic signaling and autophagy regulation). During the recovery phase, dietary GABA significantly affected plasma total protein, while temperature, dietary GABA, and their interaction significantly affected plasma cortisol. Temperature also significantly affected hepatic *hsp70α* and *hsp90βα* expression. No significant dietary GABA effects or temperature × GABA interactions were detected for any of the stress-related genes analyzed. This suggests that while temperature stress and dietary interventions can influence specific metabolic and stress-related biomarkers, their effects may be selective rather than broadly systemic. This selectivity aligns with the concept that thermodynamics constrains physiological performance and may impose novel selective pressures on temperature physiology and metabolic regulation [62]. Moreover, the relationship between GABA and stress is complicated, as it is influenced by multiple physiological and environmental factors [14,63,64], which may explain the selectivity in the biochemical variable responses observed in our biochemical analyses.

For TP, no significant main effect of temperature was observed, suggesting that acute thermal exposure alone did not markedly alter plasma protein levels during the recovery phase. However, dietary GABA significantly affected TP levels, with the GABA183 group showing a higher value than the control group. This result may indicate that dietary GABA influenced protein metabolic status during recovery from acute thermal exposure. Similar effects of dietary GABA on protein-related physiological responses have been reported in Nile tilapia and rohu [16,40]. However, because protein turnover markers, amino acid flux, or related protein synthesis and degradation pathways were not directly measured, further mechanistic studies are needed to confirm whether GABA directly enhances protein synthesis or reduces protein degradation.

Cortisol is a major endocrine indicator of the physiological stress response in fish and plays an important role in energy mobilization and adaptation to environmental challenges, including temperature stress [65,66]. Cortisol dynamics during stress and recovery can vary depending on stressor intensity, exposure duration, and recovery status, and prolonged or repeated stress may alter HPI-axis responsiveness in fish [67,68,69]. In the present study, plasma cortisol measured after a 2 h recovery period was significantly affected by temperature, dietary GABA, and their interaction. The highest mean concentration occurred in fish fed GABA218 following exposure to 20 °C, although this group did not differ significantly from the GABA183 group exposed to 20 °C. These findings demonstrate differences in recovery-phase plasma cortisol concentrations but do not establish the underlying endocrine mechanism. Higher cortisol concentrations could reflect a stronger stress response, delayed recovery, or altered cortisol clearance. Because cortisol was measured at only one recovery time point, these explanations cannot be distinguished, and the results do not demonstrate improved thermal tolerance or a faster recovery rate. The absence of pre-exposure cortisol measurements further limits interpretation of these differences. Moreover, the provisional dietary GABA range estimated from weight gain applies specifically to growth under the feeding-trial conditions and does not establish that the higher cortisol response was adaptive. Therefore, measurements before exposure, during exposure, and at multiple recovery time points are needed to clarify the cortisol response.

Furthermore, at the molecular level, hepatic *hsp70α* and *hsp90βα* expression was significantly affected by temperature, with lower mean expression in fish previously exposed to 20 °C than in fish maintained at 15 °C. Hsp70 and Hsp90 are molecular chaperones involved in protein folding, stabilization, and remodeling [70,71], and heat shock proteins are closely associated with thermal stress responses in aquatic species [72]. However, expression was measured only after a 2 h recovery period, preventing determination of whether the observed differences reflect changes during exposure, recovery-phase regulation, or other temporal responses. The transcript measurements also do not establish changes in chaperone protein abundance or impaired protein homeostasis [73]. Further time-course studies are therefore needed to characterize *hsp70α* and *hsp90βα* responses during thermal exposure and recovery. Neither dietary GABA nor its interaction with temperature significantly affected any of the stress-related genes analyzed. The thermal exposure test included only three biological replicates per diet–temperature combination. This limited replication, together with variability in the expression measurements, may have reduced sensitivity to dietary effects and temperature × GABA interactions. Accordingly, nonsignificant findings do not establish the absence of biologically relevant effects. Future studies should include greater biological replication, with sample sizes determined prospectively to detect biologically meaningful effects, particularly interactions between dietary GABA and temperature. Although GABA concentrations were measured in the finished diets, processing recovery and storage stability were not directly evaluated. Therefore, the extent of any GABA loss during diet preparation or storage remains uncertain.

## 5. Conclusions

This study demonstrated that dietary γ-aminobutyric acid (GABA) supplementation improved growth performance and feed utilization in juvenile rainbow trout. Fish fed higher dietary GABA levels showed increased final body weight, weight gain, specific growth rate, feed efficiency, and protein efficiency ratio, with the provisional model-based dietary GABA level for weight gain estimated to be approximately 198–210 mg/kg diet based on broken-line regression models. Dietary GABA had limited effects on whole-body proximate composition, plasma biochemical parameters, and free amino acid profiles under normal rearing conditions, although moisture content and plasma taurine showed some dietary differences without a clear dose-dependent response. During the recovery phase after acute thermal exposure, dietary GABA influenced plasma total protein and cortisol concentrations. In particular, cortisol was significantly affected by temperature, dietary GABA, and their interaction, demonstrating differences in plasma cortisol concentrations at the measured recovery time point. However, because cortisol was measured at a single post-recovery time point, the present data cannot distinguish a stronger stress response, delayed recovery, or altered cortisol clearance and do not establish improved thermal tolerance or a faster recovery rate. In addition, although plasma total protein was affected by dietary GABA, protein turnover markers were not measured; therefore, the observed change should be interpreted as an alteration in protein metabolic status rather than direct evidence of increased protein synthesis or reduced protein degradation. At the molecular level, hepatic *hsp70α* and *hsp90βα* expression was significantly affected by temperature, with lower mean expression following 20 °C exposure and recovery than in fish maintained at 15 °C. Neither dietary GABA nor the temperature × GABA interaction significantly affected the stress-related genes analyzed. Overall, dietary GABA supplementation improved growth performance and feed utilization and affected selected physiological parameters during the recovery phase following acute thermal exposure in juvenile rainbow trout. Protein turnover, key molecular components of the HPI axis, thermal tolerance, and survival outcomes following thermal exposure were not assessed. Therefore, these findings do not establish direct modulation of the HPI axis, improved thermal tolerance, or faster recovery from heat stress. Although growth benefits were demonstrated, the functional significance of the observed recovery-phase physiological responses remains to be clarified. Field validation and longer-term studies under chronic or fluctuating thermal conditions, including assessment of survival and multiple sampling time points, are needed before practical benefits under thermal stress can be established.

## Figures and Tables

**Table 1:** Formulation and proximate composition of the five experimental diets supplemented with different levels of γ-aminobutyric acid (GABA) for juvenile rainbow trout.

Ingredients	Diets (g/kg Dry Matter) ^1^				
	GABA_68_	GABA_108_	GABA_149_	GABA_183_	GABA_218_
Fish meal (anchovy)	250	250	250	250	250
Squid liver powder	57	57	57	57	57
Soybean meal	51	51	51	51	51
Poultry by-product	58	58	58	58	58
Tankage meal	40	40	40	40	40
Soy protein concentrate	110	110	110	110	110
Wheat flour	240	240	240	240	240
Fish oil	142	142	142	142	142
Mono calcium phosphate	10	10	10	10	10
Mineral premixture ^2^	12	12	12	12	12
Vitamin premixture ^3^	12	12	12	12	12
Vitamin C	2	2	2	2	2
Choline	6	6	6	6	6
α-cellulose	10	7.5	5	2.5	0
GABA premixture ^4^	0	2.5	5	7.5	10
Total	1000	1000	1000	1000	1000
Actual GABA concentration in the diet (mg/kg diet) ^5^	67.7	108.0	149.1	183.1	217.8
Proximate composition (% of dry matter basis) ^5^					
Moisture	8.4	8.3	7.4	8.1	8.2
Crude protein	45.3	44.6	43.9	44.5	44.0
Crude lipid	19.8	20.0	19.5	20.0	19.8
Crude ash	11.1	10.7	11.0	10.5	10.8

^1^ Diet names reflect the level of supplemental GABA added to the diet. ^2^ Mineral premix (as mg kg in diets): NaCl, 437; MgSO_4_·7H_2_O, 1380; NaH_2_PO_4_·2H_2_O, 878; Ca(H_2_PO_4_) 2H_2_O, 1367; KH_2_PO_4_, 2414; ZnSO_4_·7H_2_O, 66; Fe-Citrate, 299; Ca-lactate, 3004; MnSO_4_, 0.016; FeSO_4_, 0.0378; CuSO_4_, 0.00033; Calcium iodate, 0.0006; MgO, 0.00135; NaSeO_3_, 0.00025. ^3^ Vitamin premix (as mg/kg in diets): Ascorbic acid, 300; dl-Calcium pantothenate, 150; Choline bitartrate, 3000; Inositol, 150; Menadione, 6; Niacin, 150; Pyridoxine·HCl, 15; Riboflavin, 30; Thiamine mononitrate, 15; dl-α-tocopherol acetate, 201; Retinyl acetate, 6; Biotin, 1.5; Folic acid, 5.4; B_12_, 0.06. ^4^ Formulated by adding 4 g of >99% purity GABA to 196 g of α-cellulose (20,000 mg/kg). ^5^ Values are mean of duplicate samples.

**Table 2:** List of primers used for liver and brain gene expression.

Gene Name	Gene Symbol	Nucleotide Sequences (5′–3′)	GenBank Accession No.	Amplicon Size (bp)	Ann. Temp (Tm °C)	References
Beta-actin/Housekeeping gene	*β-actin*	F: TTCAACACCCCTGCCATGTA R: ACGGCCAGAGGCGTATAGG	AF157514	63	57.3 61.0	[21]
Growth hormone receptor-I	*ghr-I*	F: CCTGACCTACTGGGCAACAT R: CTCAGGCACTGAATTCCCAT	NM_001124535	105	59.3 57.3	[22]
Growth hormone receptor-II	*ghr-II*	F: CATGGCAACTTCCCACATTCT R: GCTCCTGCGACACAACTGTTAG	NM_001124731	65	57.9 62.1	[23]
Insulin-like growth factor-I	*igf-I*	F: TGCGGAGAGAGAGGCTTTTA R: AGCACTCGTCCACAATACCA	NM_001124696.1	91	57.3 57.3	[24,25]
Insulin-like growth factor-II	*igf-II*	F: CATGGTGCCCACACTCAAAC R: CTTCCTCTGCCACACCTCATA	NM_001124697.1	82	59.3 59.8	[26]
Growth hormone 1	*gh1*	F: GGTTCGGTTTCCCAGATACAGA R: TGAAAAGCATGTTCAATGGAGAA	NM_001124689.1	85	60.3 55.3	[27,28]
Growth hormone 2	*gh2*	F: GGAGCGGAGCAGCAAGAG R: TGCAGGGAAAGGTCTCATCTG	NM_001124690.2	68	60.5 59.8	[28]
Gamma-aminobutyric acid type A receptor-associated protein-like 1	*gabarapl1*	F: GTGGAAAAAGCCCCCAAAGC R: CCTCTTCATGGTGCTCCTGGTA	CA345480	208	59.3 62.1	[29]
Warm temperature acclimation-related 65 kDa protein-1	*wap65-1*	F: ACATGAGGGACACGACCATG R: AAACCCTTGAACACATGGCC	XM_036975307	135	59.3 57.3	[30]
Warm temperature acclimation-related 65 kDa protein-2	*wap65-2*	F: ACCCTAAAAGCCTGAATGCG R: TGGCAGATAGGTCAATGTCCAG	XM_021608647	126	57.3 60.3	[30]
Heat-shock protein 90βα	*hsp90βα*	F: AATGGGTAACCTGGTCAGTG R: CTGAATACAGACAGGTCTGA	AB196457	300	57.1 53.8	[31]
Heat-shock protein 70α	*hsp70α*	F: ACCACGAAATTGGGGAGAAA R: TGCAATGTCCAACAATGAAA	AB176854	213	57.3 54.3	[31]

**Table 3:** Growth performance of juvenile rainbow trout fed the experimental diets for eight weeks ^1^.

Parameters ^3^	Diets ^2^					*p*-Value	Linear	Quadratic
	GABA_68_	GABA108	GABA_149_	GABA_183_	GABA_218_			
IBW (g)	18.9 ± 0.1 ^ns^	19.0 ± 0.2	19.0 ± 0.3	18.8 ± 0.3	18.8 ± 0.2	0.5640	0.2594	0.5247
FBW (g)	52.9 ± 0.6 ^c^	53.9 ± 0.6 ^bc^	56.0 ± 0.7 ^a^	55.8 ± 1.3 ^ab^	57.0 ± 0.4 ^a^	0.0004	<0.0001	0.2438
WG (%)	179.0 ± 1 ^b^	184.0 ± 1 ^b^	195.0 ± 5 ^ab^	198.0 ± 1 ^ab^	203.0 ± 2 ^a^	0.0036	0.0004	0.2280
SGR (%)	1.83 ± 0.00 ^b^	1.86 ± 0.02 ^b^	1.93 ± 0.03 ^ab^	1.95 ± 0.04 ^ab^	1.98 ± 0.01 ^a^	0.0096	0.0007	0.7006
FE (%)	94.9 ± 0.7 ^b^	96.5 ± 0.6 ^ab^	101 ± 2 ^ab^	101 ± 1 ^ab^	103 ± 2 ^a^	0.0090	0.0006	0.6756
PER	2.09 ± 0.02 ^c^	2.16 ± 0.03 ^bc^	2.31 ± 0.03 ^ab^	2.28 ± 0.05 ^ab^	2.34 ± 0.03 ^a^	0.0237	0.0023	0.4854
SR (%)	100.0 ± 0 ^ns^	100.0 ± 0	100.0 ± 0	100.0 ± 0	97.8 ± 2.2	0.4516	0.1877	0.2596
CF	1.05 ± 0.01 ^ns^	1.05 ± 0.01	1.08 ± 0.01	1.05 ± 0.01	1.07 ± 0.03	0.6656	0.6960	0.6031
HSI (%)	1.03 ± 0.03 ^ns^	1.05 ± 0.01	1.03 ± 0.02	1.05 ± 0.01	0.99 ± 0.02	0.4043	0.1940	0.6364
VSI (%)	9.16 ± 0.62 ^ns^	9.76 ± 0.57	9.62 ± 0.26	9.69 ± 0.19	10.3 ± 0.2	0.4453	0.1145	0.8450

^1^ Values are means from triplicate groups of fish where the values in each row with different superscripts are significantly different (±SEM; *p* < 0.05); ns, not significant (*p* > 0.05). ^2^ Diet names reflect the level of supplemental GABA added to the diet. ^3^ IBW: initial body weight (g); FBW: final body weight (g); WG: weight gain (%); SGR: specific growth rate (% day^−1^); FE: feed efficiency (%); PER: protein efficiency ratio; SR: survival rate (%); CF: condition factor; HSI: hepatosomatic index (%); VSI: viscerosomatic index (%).

**Table 4:** Whole-body proximate compositions (% of as-is basis) of juvenile rainbow trout fed the experimental diets for 8 weeks ^1^.

Parameters	Diets ^2^					*p*-Value	Linear	Quadratic
	GABA_68_	GABA108	GABA_149_	GABA_183_	GABA_218_			
Moisture	71.6 ± 0.2 ^a^	70.6 ± 0.4 ^ab^	69.1 ± 0.9 ^ab^	69.0 ± 0.4 ^b^	69.5 ± 0.6 ^ab^	0.0338	0.0081	0.0607
Crude protein	15.6 ± 0.2 ^ns^	16.7 ± 0.2	18.2 ± 1.3	17.3 ± 0.2	16.7 ± 0.1	0.2262	0.5253	0.0611
Crude lipid	10.0 ± 0.1 ^ns^	9.89 ± 0.49	9.80 ± 0.55	11.0 ± 0.2	11.2 ± 0.4	0.0891	0.0200	0.2586
Crude ash	2.35 ± 0.03 ^ns^	2.60 ± 0.15	2.57 ± 0.08	2.40 ± 0.03	2.35 ± 0.11	0.0927	0.1665	0.0222

^1^ Values are means from triplicate groups of fish where the values in each row with different superscripts are significantly different (±SEM; *p* < 0.05); ns, not significant (*p* > 0.05). ^2^ Diet names reflect the level of supplemental GABA added to the diet.

**Table 5:** Plasma metabolites of juvenile rainbow trout fed the experimental diets for 8 weeks ^1^.

Parameters ^3^	Diets ^2^					*p*-Value	Linear	Quadratic
	GABA_68_	GABA_108_	GABA_149_	GABA_183_	GABA_218_			
GOT	203 ± 71 ^ns^	267 ± 48	387 ± 121	331 ± 13	285 ± 68	0.4999	0.3482	0.1760
GPT	14.7 ± 0.3 ^ns^	27.3 ± 2.4	27.3 ± 5.5	25.3 ± 1.3	23.3 ± 0.7	0.0539	0.1087	0.0124
TP	2.97 ± 0.15 ^ns^	3.27 ± 0.13	3.33 ± 0.41	3.6 ± 0.12	3.33 ± 0.33	0.5633	0.2171	0.3529
TG	283 ± 26 ^ns^	306 ± 38	337 ± 47	446 ± 67	385 ± 47	0.1822	0.0433	0.6143
TCHO	226 ± 9 ^ns^	181 ± 3	199 ± 28	203 ± 12	203 ± 18	0.4749	0.6499	0.2468
GLU	72.3 ± 2.7 ^ns^	78 ± 13.9	64 ± 11.4	70.0 ± 4.6	67.3 ± 10.5	0.8684	0.5659	0.9278

^1^ Values are presented as means ± SEM from three replicate groups of fish; ns, not significant (*p* > 0.05). ^2^ Diet names reflect the level of supplemental GABA added to the diet. ^3^ GOT: glutamic oxaloacetic transaminase (U/L); GPT: glutamic pyruvic transaminase (U/L); TP: total protein (g/dL); TG: triglyceride (mg/dL); TCHO: total cholesterol (mg/dL); GLU: glucose (mg/dL).

**Table 6:** Plasma-free amino acid profile (% of as-is basis) of rainbow trout fed the experimental diets for 8 weeks ^1^.

Amino Acid	Diets ^2^					*p*-Value	Linear	Quadratic
	GABA_68_	GABA_108_	GABA_149_	GABA_183_	GABA_218_			
Phosphoserine	2.41 ± 0.28 ^ns^	2.26 ± 0.09	2.04 ± 0.10	2.12 ± 0.14	2.17 ± 0.11	0.5725	0.2482	0.2822
Taurine	85.4 ± 1.3 ^b^	114.0 ± 10 ^a^	82.2 ± 2.6 ^b^	101.0 ± 6 ^ab^	91.6 ± 6.2 ^ab^	0.0224	0.9795	0.2840
Hydroxyproline	15.0 ± 2.3 ^ns^	12.9 ± 1.9	15.8 ± 1.1	17.8 ± 0.7	15.0 ± 1.6	0.3789	0.3613	0.7087
Threonine	17.9 ± 1.1 ^ns^	16.5 ± 0.5	18.8 ± 0.5	18.0 ± 1.5	16.7 ± 1.4	0.5671	0.8356	0.5127
Serine	18.0 ± 0.3 ^ns^	14.8 ± 1.1	17.5 ± 0.9	19.1 ± 1.4	17.1 ± 1.4	0.1585	0.4778	0.7662
Asparagine	21.9 ± 1.4 ^ns^	16.8 ± 1.9	23.5 ± 1.4	22.0 ± 2.9	22.0 ± 2.3	0.2473	0.4165	0.8057
Glutamic acid	48.1 ± 2.2 ^ns^	42.3 ± 4.3	48.8 ± 2.5	50.3 ± 2.7	47.1 ± 3.6	0.4934	0.5753	0.9969
Proline	3.39 ± 0.35 ^ns^	3.23 ± 0.44	2.76 ± 0.11	3.29 ± 0.08	3.03 ± 0.44	0.6668	0.5262	0.5314
Glycine	39.0 ± 2.4 ^ns^	34.6 ± 4.4	36.2 ± 4.5	43.7 ± 2.6	34.9 ± 2.8	0.3725	0.9385	0.8216
Alanine	31.4 ± 0.7 ^ns^	32.1 ± 6.2	33.2 ± 4.7	38.1 ± 2.2	31.9 ± 3.1	0.7432	0.5869	0.5127
α-amino-n-butyric acid	7.01 ± 0.40 ^ns^	6.04 ± 0.47	4.80 ± 2.40	5.23 ± 2.63	5.90 ± 0.66	0.8937	0.5720	0.4390
Valine	29.9 ± 2.8 ^ns^	26.9 ± 0.2	31.6 ± 2.5	29.5 ± 1.2	27.3 ± 1.6	0.4376	0.6478	0.4838
Cystine	1.51 ± 0.09 ^ns^	1.33 ± 0.13	1.38 ± 0.04	1.44 ± 0.09	1.17 ± 0.11	0.2072	0.0886	0.6275
Methionine	10.4 ± 0.3 ^ns^	10.0 ± 0.5	10.4 ± 0.0	10.3 ± 0.4	9.40 ± 0.7	0.4520	0.2348	0.3657
Isoleucine	14.3 ± 1.4 ^ns^	13.5 ± 0.4	14.5 ± 2.1	14.4 ± 0.7	13.1 ± 0.6	0.8975	0.7008	0.6771
Leucine	22.3 ± 2.1 ^ns^	21.3 ± 0.6	23.2 ± 2.6	22.3 ± 0.8	21.6 ± 0.9	0.9290	0.9294	0.7059
Tyrosine	9.04 ± 0.26 ^ns^	9.51 ± 0.47	9.10 ± 0.32	9.82 ± 0.33	9.42 ± 0.51	0.6236	0.4066	0.6931
Phenylalanine	11.0 ± 0.3 ^ns^	12.5 ± 0.7	12.5 ± 0.4	12.6 ± 0.4	12.1 ± 0.6	0.2053	0.1481	0.0682
γ-aminobutyric acid	18.6 ± 0.9 ^ns^	16.6 ± 1.9	18.7 ± 0.4	16.9 ± 1.9	18.7 ± 3.9	0.9085	0.9498	0.6484
Histidine	22.9 ± 1.3 ^ns^	20.4 ± 0.0	23.4 ± 1.7	23.8 ± 1.4	22.4 ± 2.3	0.5638	0.6198	0.9243
Tryptophan	1.37 ± 0.68 ^ns^	0.72 ± 0.72	1.60 ± 0.33	1.95 ± 0.14	2.24 ± 0.41	0.3209	0.0909	0.4869
Ornithine	3.59 ± 0.63 ^ns^	1.23 ± 1.23	4.35 ± 0.67	3.29 ± 0.43	3.04 ± 0.67	0.1392	0.7031	0.9910
Lysine	3.86 ± 0.54 ^ns^	3.44 ± 0.18	4.22 ± 0.32	3.49 ± 0.22	3.61 ± 0.47	0.5938	0.7089	0.7661
Ammonia	11.0 ± 0.7 ^ns^	9.30 ± 0.2	10.1 ± 0.8	8.90 ± 0.9	9.60 ± 0.4	0.2540	0.1506	0.2982
Arginine	54.7 ± 1.7 ^ns^	57.2 ± 6.5	54.9 ± 2.9	52.9 ± 0.6	57.4 ± 6.9	0.9460	0.9297	0.8034
Total amino acid	501 ± 7 ^ns^	497 ± 34	503 ± 30	530 ± 14	496 ± 37	0.8907	0.8027	0.7103

^1^ Values are means from triplicate groups of fish where the values in each row with different superscripts are significantly different (±SEM; *p* < 0.05); ns, not significant (*p* > 0.05). ^2^ Diet names reflect the level of supplemental GABA added to the diet.

**Table 7:** Brain-free amino acid profile (% of as-is basis) of rainbow trout fed the experimental diets for 8 weeks ^1^.

Amino Acid	Diets ^2^					*p*-Value	Linear	Quadratic
	GABA_68_	GABA_108_	GABA_149_	GABA_183_	GABA_218_			
Phosphoserine	248.0 ± 10 ^ns^	303.0 ± 4	272.0 ± 20	327.0 ± 55	249.0 ± 23	0.2802	0.3556	0.5116
Taurine	4599.0 ± 215 ^ns^	5867.0 ± 148	4730.0 ± 475	6532.0 ± 1319	4553.0 ± 636	0.2384	0.3103	0.5997
Phosphoethanolamine	215.0 ± 10 ^ns^	251.0 ± 7	202.0 ± 15	260.0 ± 49	173.0 ± 12	0.1405	0.5733	0.6847
Aspartic Acid	435.0 ± 22 ^ns^	515.0 ± 3	528.0 ± 138	574.0 ± 91	420.0 ± 57	0.6208	0.8365	0.5215
Hydroxyproline	63.0 ± 14.2 ^ns^	85.2 ± 11.7	57.9 ± 9.3	90.8 ± 15.2	65.5 ± 16.0	0.3825	0.2503	0.8696
Threonine	210.0 ± 16 ^ns^	258.0 ± 5	213.0 ± 19	290.0 ± 62	186.0 ± 25	0.2774	0.5095	0.7145
Serine	242.0 ± 12 ^ns^	280.0 ± 23	226.0 ± 21	335.0 ± 54	235.0 ± 24	0.1416	0.3921	0.4628
Glutamic Acid	3808.0 ± 198 ^ns^	4385.0 ± 42	3872.0 ± 386	5157.0 ± 826	3666.0 ± 500	0.2337	0.5412	0.4275
Proline	27.4 ± 1.2 ^ns^	32.0 ± 3.0	26.7 ± 3.8	33.6 ± 4.9	27.4 ± 2.6	0.5027	0.3642	0.8494
Glycine	330.0 ± 19 ^ns^	422.0 ± 30	313.0 ± 32	494.0 ± 101	355.0 ± 39	0.1710	0.2051	0.4557
Alanine	164 ± 7 ^ns^	209 ± 2	168 ± 22	215 ± 32	170 ± 18	0.2480	0.1663	0.7727
Citrulline	0.00 ± 0 ^ns^	0.00 ± 0	6.44 ± 6.44	0.00 ± 0	0.00 ± 0	0.4516	0.4956	0.5634
Valine	46.8 ± 3.2 ^ns^	58.1 ± 6.8	44.6 ± 4.9	53.6 ± 10.0	49.6 ± 5.3	0.6097	0.2073	0.7352
Cystine	69.4 ± 5.6 ^ns^	83.4 ± 2.5	67.3 ± 8.6	77.2 ± 13.6	61.5 ± 5.4	0.3970	0.4050	0.4801
Methionine	15.6 ± 1.4 ^ns^	18.2 ± 2.7	13.0 ± 0.9	21.9 ± 3.8	13.9 ± 1.9	0.1335	0.4391	0.7247
Isoleucine	34.6 ± 2.7 ^ns^	42.0 ± 3.8	37.2 ± 3.4	42.5 ± 6.6	36.0 ± 2.6	0.5712	0.3238	0.7454
Leucine	51.8 ± 3.0 ^ns^	62.6 ± 6.5	54.8 ± 6.4	59.0 ± 8.1	53.9 ± 2.1	0.6855	0.2763	0.9181
Tyrosine	35.4 ± 3.9 ^ns^	44.9 ± 1.8	35.2 ± 4.6	42.7 ± 7.7	35.2 ± 4.1	0.4595	0.2375	0.7860
Phenylalanine	50.2 ± 3.8 ^ns^	62.1 ± 2.7	50.6 ± 6.9	60.2 ± 11.6	49.9 ± 6.0	0.5766	0.3158	0.8884
β-aminoisobutyric acid	0.00 ± 0 ^ns^	0.00 ± 0	77.0 ± 77	295.0 ± 295	0.00 ± 0	0.5109	0.8613	0.2202
γ-aminobutyric acid	1259.0 ± 111 ^ns^	1611.0 ± 105	1313.0 ± 145	1486.0 ± 200	1203.0 ± 93	0.2606	0.1977	0.6312
Histidine	1575.0 ± 87 ^ns^	1660.0 ± 145	1634.0 ± 277	1624.0 ± 212	1578.0 ± 22	0.9955	0.8413	0.9881
Tryptophan	116.0 ± 2 ^ns^	145.0 ± 7	139.0 ± 19	175.0 ± 27	117.0 ± 15	0.1485	0.5178	0.2051
Lysine	432.0 ± 18 ^ns^	557.0 ± 11	432.0 ± 50	469.0 ± 62	438.0 ± 26	0.1860	0.0624	0.2729

^1^ Values are presented as means ± SEM from three replicate groups of fish; ns, not significant (*p* > 0.05). ^2^ Diet names reflect the level of supplemental GABA added to the diet.

**Table 8:** Relative gene expression levels ^1^ in the brain of juvenile rainbow trout fed the experimental diets for 8 weeks ^2^.

Genes ^4^	Diets ^3^					*p*-Value
	GABA_68_	GABA_108_	GABA_149_	GABA_183_	GABA_218_	
*gh1*	1.19 ± 0.52 ^ns^	20.9 ± 14.9	9.12 ± 7.86	12.1 ± 6.4	1.70 ± 0.55	0.4104
*gh2*	2.43 ± 1.98 ^ns^	15.9 ± 10.5	3.74 ± 3.4	0.79 ± 0.18	0.35 ± 0.13	0.3749
*gabarapl1*	1.13 ± 0.41 ^ns^	0.31 ± 0.08	3.55 ± 3.28	0.65 ± 0.44	1.65 ± 0.45	0.7340

^1^ Relative gene express level (GABA_68_ was set as a control; *β-actin* used as housekeeping gene). ^2^ Values are presented as means ± SEM from three replicate groups of fish; ns, not significant (*p* > 0.05). ^3^ Diet names reflect the level of supplemental GABA added to the diet. ^4^ *gh1*: growth hormone 1; *gh2*: growth hormone 2; *gabarapl1*: gamma-aminobutyric acid type A receptor-associated protein-like 1.

**Table 9:** Relative gene expression levels ^1^ in the liver of juvenile rainbow trout fed the experimental diets for 8 weeks ^2^.

Genes ^4^	Diets ^3^					*p*-Value
	GABA_68_	GABA_108_	GABA_149_	GABA_183_	GABA_218_	
*ghr-* *I*	1.09 ± 0.33 ^ns^	0.27 ± 0.19	0.69 ± 0.06	0.46 ± 0.12	1.40 ± 0.52	0.1169
*ghr-* *II*	1.11 ± 0.37 ^a^	0.15 ± 0.1 ^b^	0.98 ± 0.07 ^ab^	0.57 ± 0.20 ^ab^	1.29 ± 0.09 ^a^	0.0162
*igf-* *I*	1.21 ± 0.49 ^ab^	0.08 ± 0.04 ^b^	1.07 ± 0.13 ^ab^	0.78 ± 0.22 ^ab^	1.38 ± 0.24 ^a^	0.0488
*igf-* *II*	1.18 ± 0.4 ^ns^	0.22 ± 0.14	0.74 ± 0.3	0.33 ± 0.08	1.38 ± 0.26	0.0505

^1^ Relative gene express level (GABA_68_ was set as a control; *β-actin* used as housekeeping gene). ^2^ Values are means from triplicate groups of fish where the values in each row with different superscripts are significantly different (±SEM; *p* < 0.05); ns, not significant (*p* > 0.05). ^3^ Diet names reflect the level of supplemental GABA added to the diet. ^4^ *ghr-I*: growth hormone receptor-I; *ghr-II:* growth hormone receptor-II; *igf-I:* insulin-like growth factor-I; *igf-II*: insulin-like growth factor-II.

**Table 10:** Plasma metabolites and cortisol response of rainbow trout after the acute thermal exposure (20 °C) and recovery test ^1^.

				Parameters ^2^			
	GOT	GPT	TP	TG	TCHO	GLU	Cortisol ^3^
Interactive effect between temperature and GABA							
T_15_GABA_68_	322 ± 54 ^ns^	26.7 ± 3.8 ^ns^	2.70 ± 0.09 ^ns^	335 ± 18 ^ns^	198 ± 20 ^ns^	80.5 ± 10.2 ^ns^	3.87 ± 0.29 ^d^
T_15_GABA_108_	458 ± 83	36.0 ± 6.8	3.13 ± 0.27	305 ± 36	201 ± 14	116 ± 7	5.37 ± 0.29 ^cd^
T_15_GABA_149_	356 ± 35	27.7 ± 2.3	3.07 ± 0.08	410 ± 45	205 ± 9	104 ± 13	6.32 ± 0.55 ^bcd^
T_15_GABA_183_	408 ± 28	26.7 ± 2.2	3.40 ± 0.09	344 ± 20	201 ± 10	107 ± 10	7.60 ± 0.45 ^bc^
T_15_GABA_218_	356 ± 53	25.0 ± 2.7	3.00 ± 0.16	336 ± 35	207 ± 8	127 ± 16	6.24 ± 0.22 ^bcd^
T_20_GABA_68_	307 ± 30	66.0 ± 40.8	3.10 ± 0.12	272 ± 17	209 ± 13	86.3 ± 5.4	7.27 ± 0.35 ^bcd^
T_20_GABA_108_	339 ± 32	60.0 ± 30.8	3.17 ± 0.10	266 ± 28	202 ± 14	65.2 ± 5.1	7.29 ± 0.42 ^bcd^
T_20_GABA_149_	397 ± 81	31.6 ± 2.4	2.88 ± 0.19	337 ± 15	205 ± 14	67.3 ± 12.6	8.07 ± 1.23 ^bc^
T_20_GABA_183_	350 ± 36	48.7 ± 23.1	3.33 ± 0.19	454 ± 80	288 ± 36	131 ± 32	9.63 ± 1.51 ^ab^
T_20_GABA_218_	467 ± 68	31.2 ± 3.0	3.20 ± 0.13	306 ± 18	232 ± 17	121 ± 19	12.4 ± 1.23 ^a^
Main effect of temperature							
T_15_	380 ± 24 ^ns^	28.4 ± 1.9 ^ns^	3.06 ± 0.11 ^ns^	346 ± 17 ^ns^	202 ± 2 ^ns^	107 ± 8 ^ns^	5.88 ± 0.06 ^b^
T_20_	372 ± 28	47.5 ± 7.1	3.14 ± 0.07	327 ± 34	227 ± 16	94.2 ± 13.6	8.93 ± 0.24 ^a^
Main effect of GABA							
GABA_68_	314 ± 8 ^ns^	46.3 ± 19.7 ^ns^	2.90 ± 0.20 ^b^	304 ± 31 ^ns^	204 ± 5 ^ns^	83.4 ± 2.9 ^ns^	5.57 ± 0.03 ^b^
GABA_108_	398 ± 59	48.0 ± 12.0	3.15 ± 0.02 ^ab^	286 ± 20	201 ± 0	90.5 ± 25.3	6.33 ± 0.07 ^b^
GABA_149_	377 ± 20	29.6 ± 2.0	2.97 ± 0.09 ^ab^	373 ± 36	205 ± 0	85.6 ± 18.3	7.19 ± 0.34 ^ab^
GABA_183_	379 ± 29	37.7 ± 11.0	3.37 ± 0.03 ^a^	399 ± 55	244 ± 44	119 ± 12	8.61 ± 0.53 ^a^
GABA_218_	412 ± 55	28.1 ± 3.1	3.10 ± 0.10 ^ab^	321 ± 15	219 ± 13	124 ± 3	9.32 ± 0.50 ^a^
Two-way ANOVA (*p*-value)							
Temperature	0.8142	0.1135	0.4346	0.4146	0.0575	0.1858	<0.0001
GABA	0.4476	0.7521	0.0330	0.0548	0.0857	0.1610	<0.0001
Temperature × GABA	0.2771	0.8785	0.3495	0.0993	0.0809	0.0949	0.0387

^1^ Values are means from triplicate groups of fish where the values in each row with different superscripts are significantly different (±SEM; *p* < 0.05); ns, not significant (*p* > 0.05). ^2^ GOT: glutamic oxaloacetic transaminase (U/L); GPT: glutamic pyruvic transaminase (U/L); TP: total protein (g/dL); TG: triglyceride (mg/dL); TCHO: total cholesterol (mg/dL); GLU: glucose (mg/dL); ^3^ cortisol (ng/mL).

**Table 11:** Relative gene expression levels in the liver after the acute thermal exposure (20 °C) and recovery test ^1^.

	Genes ^2^				
	*wap65-1*	*wap65-2*	*hsp70α*	*hsp90βα*	*gabarapl1*
Interactive effect between temperature and GABA					
T_15_GABA_68_	1.1 ± 0.19 ^ns^	1.02 ± 0.1 ^ns^	1.27 ± 0.3 ^ns^	1.31 ± 0.32 ^ns^	1.11 ± 0.24 ^ns^
T_15_GABA_108_	1.58 ± 0.62	1.51 ± 0.58	5.19 ± 1.56	7.48 ± 2.37	3.12 ± 1.27
T_15_GABA_149_	1.73 ± 1.09	1.1 ± 0.67	9.69 ± 3.61	8.73 ± 2.77	3.67 ± 1.99
T_15_GABA_183_	0.83 ± 0.08	0.39 ± 0.06	11.0 ± 3.05	12.3 ± 3.35	1.35 ± 0.41
T_15_GABA_218_	0.88 ± 0.3	0.68 ± 0.3	13.88 ± 6.41	16.62 ± 7.68	1.71 ± 0.55
T_20_GABA_68_	1.12 ± 0.25	1.3 ± 0.44	1.37 ± 0.4	1.08 ± 0.18	2.98 ± 1.98
T_20_GABA_108_	0.55 ± 0.15	0.75 ± 0.17	9.37 ± 4.82	2.83 ± 0.85	2.71 ± 0.97
T_20_GABA_149_	0.4 ± 0.06	0.58 ± 0.11	1.97 ± 1.07	1.11 ± 0.29	3.16 ± 1.01
T_20_GABA_183_	0.65 ± 0.13	0.61 ± 0.18	2.92 ± 1.56	3.97 ± 2.69	3.62 ± 1.19
T_20_GABA_218_	0.67 ± 0.21	0.82 ± 0.22	2.04 ± 0.82	1.11 ± 0.11	3.29 ± 0.79
Main effect of temperature					
T_15_	1.22 ± 0.18 ^ns^	0.94 ± 0.19 ^ns^	8.2 ± 2.23 ^a^	9.3 ± 2.55 ^a^	2.19 ± 0.51 ^ns^
T_20_	0.68 ± 0.12	0.81 ± 0.13	3.5 ± 1.48 ^b^	2.02 ± 0.59 ^b^	3.2 ± 0.15
Main effect of GABA					
GABA_68_	1.11 ± 0.01 ^ns^	1.16 ± 0.14 ^ns^	1.32 ± 0.05 ^ns^	1.19 ± 0.12 ^ns^	2.05 ± 0.94 ^ns^
GABA_108_	1.06 ± 0.52	1.13 ± 0.38	7.28 ± 2.09	5.15 ± 2.32	2.91 ± 0.21
GABA_149_	1.07 ± 0.66	0.84 ± 0.26	5.83 ± 3.86	4.9 ± 3.81	3.42 ± 0.25
GABA_183_	0.74 ± 0.09	0.5 ± 0.11	7.0 ± 4.04	8.14 ± 4.17	2.5 ± 1.14
GABA_218_	0.77 ± 0.11	0.75 ± 0.07	7.96 ± 5.92	8.86 ± 7.75	2.5 ± 0.79
Two-way ANOVA (*p*-value)					
Temperature	0.0508	0.5613	0.0195	0.0004	0.2057
GABA	0.8514	0.2976	0.2136	0.1049	0.8211
Temperature X GABA	0.4396	0.4396	0.0701	0.1622	0.6498

^1^ Relative gene expression level (T_15_GABA_68_ was set as a control; *β-actin* used as housekeeping gene). Values are presented as means ± SEM from three replicate groups of fish; ns, not significant (*p* > 0.05), the values in each row with different superscripts are significantly different (±SEM; *p* < 0.05). Statistical analyses were performed on ΔCt values, whereas relative expression is presented as 2^−ΔΔCt^ fold changes (±SEM; *p* < 0.05). ^2^ *wap65-1*: warm temperature acclimation-related 65 kDa protein-1; *wap65-2*: warm temperature acclimation-related 65 kDa protein-2; *hsp70α*: heat-shock protein 70α; *hsp90βα*: heat-shock protein 90βα; *gabarapl1*: gamma-aminobutyric acid type A receptor-associated protein-like 1.

## Data Availability

The original contributions presented in this study are included in the article. Further inquiries can be directed to the corresponding authors.

## Supplementary Materials

The following supporting information can be downloaded at: https://www.mdpi.com/article/10.3390/ani16203324/s1, Figure S1. Plasma biochemical parameters and cortisol levels in juvenile rainbow trout after the acute thermal exposure and recovery test. Box plots showing plasma glutamic oxaloacetic transaminase, glutamic pyruvic transaminase, total protein, triglyceride, total cholesterol, glucose, and cortisol levels in juvenile rainbow trout maintained at 15 °C or exposed to 20 °C for 2 h, followed by 2 h recovery at 15 °C after dietary γ-aminobutyric acid (GABA) supplementation. Blue and red boxes represent the 15 °C and 20 °C groups, respectively. Boxes indicate the interquartile range, horizontal lines indicate the median, whiskers indicate data range, and open circles represent individual observations. GOT, glutamic oxaloacetic transaminase; GPT, glutamic pyruvic transaminase; TP, total protein; TG, triglyceride; TCHO, total cholesterol; GLU, glucose. The original contributions presented in this study are included in the article. Further inquiries can be directed to the corresponding authors.
