# Supplementary material for: Dietary γ-Aminobutyric Acid (GABA) Improves Growth Performance and Increases Plasma Cortisol During Recovery from Short-Term Acute Thermal Exposure in Juvenile Rainbow Trout (Oncorhynchus mykiss)
Source: Animals (Basel). 2026 Oct 21;16(20):3324. doi: 10.3390/ani16203324 (PMC13700852; doi:10.3390/ani16203324)
Supplement: Supplementary file 1 [file animals-16-03324-s001.zip › animals-4604871-supplementary.pdf]

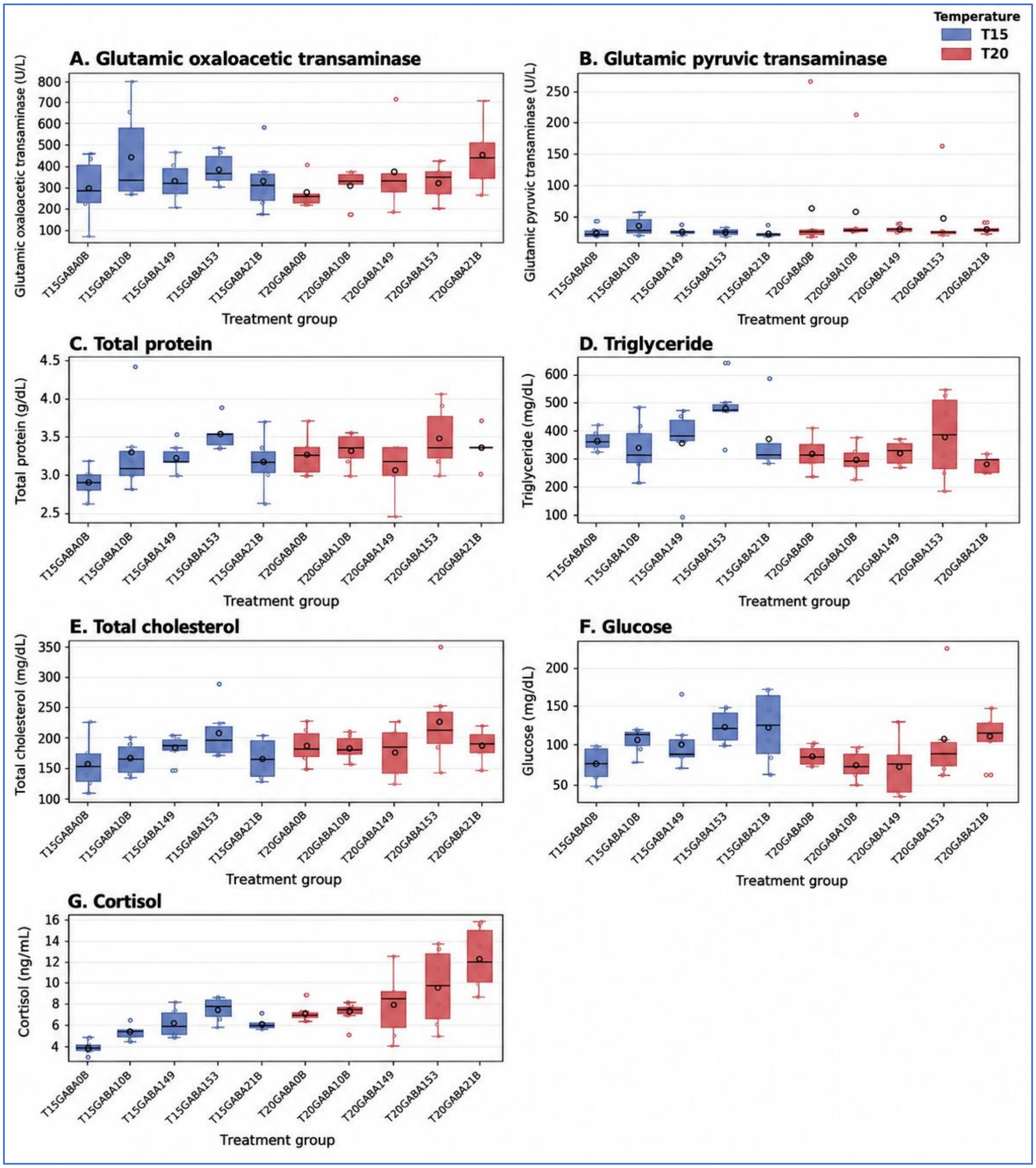

Figure S1. Plasma biochemical parameters and cortisol levels in juvenile rainbow trout after the acute thermal exposure and recovery test. Box plots showing plasma glutamic oxaloacetic transaminase, glutamic pyruvic transaminase, total protein, triglyceride, total cholesterol, glucose, and cortisol levels in juvenile rainbow trout maintained at 15°C or exposed to 20°C for 2 h, followed by 2 h recovery at 15°C after dietary γ-aminobutyric acid (GABA) supplementation. Blue and red boxes represent the 15°C and 20°C groups, respectively. Boxes indicate the interquartile range, horizontal lines indicate the median, whiskers indicate data range, and open circles represent individual observations. GOT, glutamic oxaloacetic transaminase; GPT, glutamic pyruvic transaminase; TP, total protein; TG, triglyceride; TCHO, total cholesterol; GLU, glucose.
